# Passive transfer of patient-derived anti-nephrin autoantibodies causes a podocytopathy with minimal change lesions

**DOI:** 10.1172/JCI186769

**Published:** 2025-01-16

**Authors:** Felicitas E. Hengel, Silke Dehde, Oliver Kretz, Jonas Engesser, Tom Zimmermann, Tobias B. Huber, Nicola M. Tomas

**Affiliations:** 1 III. Department of Medicine,; 2 Hamburg Center for Kidney Health (HCKH), and; 3 Department of Pathology, University Medical Center Hamburg-Eppendorf, Hamburg, Germany.

**Keywords:** Autoimmunity, Nephrology, Autoimmune diseases

**To the editor:** Autoantibodies against nephrin, a key podocyte signaling protein forming the slit diaphragm between podocyte foot processes, were recently identified in a major portion of patients with podocytopathies (1, 2). Anti-nephrin autoantibodies strongly correlate with disease activity in these patients, and nephrin-immunized mice develop anti-nephrin antibodies, alterations in nephrin phosphorylation, podocyte ultrastructure, and nephrotic syndrome, suggesting pathogenicity of these autoantibodies (1). However, a pathogenic role of human anti-nephrin autoantibodies in the development of a podocytopathy has not been demonstrated.

A 71-year-old female developed a rapid-onset nephrotic syndrome and was diagnosed with minimal change disease (MCD) by kidney biopsy (Figure 1, A and B and Supplemental Material; supplemental material available online with this article; https://doi.org/10.1172/JCI186769DS1). We found high circulating anti-nephrin autoantibody levels (1) at the time of exacerbating nephrotic syndrome and low autoantibody levels at the time of partial remission (Figure 1A, red line), illustrating the strong association of anti-nephrin autoantibodies with disease activity in patients with anti-nephrin–associated podocytopathy.

Using immunoprecipitation (IP) and subsequent Western blotting, we detected low crossreactivity of patient anti-nephrin autoantibodies with mouse, rat, and guinea pig nephrin, and higher reactivity with rabbit and pig nephrin (Figure 1C and Supplemental Figure 1A). Based on this, we chose rabbits for the transfer of human IgG purified from the plasma exchange (PLEX) fluid of the described patient or from control serum. Purified patient IgG, but not control IgG, precipitated recombinant human nephrin as well as nephrin from human and rabbit glomerular extracts (Supplemental Figure 1B). In line with this, the patient serum, PLEX fluid, and purified IgG contained high levels of anti-nephrin autoantibodies, as measured by quantitative IP/ELISA assay (Supplemental Figure 1C) (1).

Next, we transferred control and anti-nephrin IgG to one New Zealand White rabbit each. Animals were monitored daily and sacrificed at day 5. The anti-nephrin rabbit, but not the control rabbit, had detectable circulating anti-nephrin IgG (Supplemental Figure 2A). Notably, the anti-nephrin rabbit, but not the control rabbit, developed increasing proteinuria over the observation period of five days (Figure 1D). Neither the rabbit receiving anti-nephrin IgG nor the control rabbit exhibited major changes by periodic acid-Schiff (PAS) staining, such as global or segmental sclerosis, cellular proliferation or infiltration, matrix increase, or necrosis, and capillary walls were delicate (Figure 1E, left, and Supplemental Figure 2B). Further morphological workup revealed moderate podocyte foot process effacement in the absence of electron-dense deposits in the anti-nephrin rabbit by electron microscopy (Figure 1E, right) and sparse positivity for human IgG located at the glomerular filtration barrier by immunofluorescence (Figure 1F, left, and Supplemental Figure 2C, left), while these changes were absent in the control rabbit. Importantly, we did not find complement consumption in the sera of the injected rabbits (Supplemental Figure 2D), which, together with the absence of homologous rabbit IgG and complement deposition (Figure 1F and Supplemental Figure 2C, middle and right), argues against the development of serum sickness as a cause of disease in the injected rabbits.

As anti-nephrin autoantibodies were previously shown to induce nephrin phosphorylation in patients and mouse models (1, 3), we investigated target antigen phosphorylation in the injected rabbits. Nephrin tyrosine phosphorylation strongly increased in response to human anti-nephrin autoantibodies (Figure 1, G and H), demonstrating specific and direct anti-nephrin antibody–mediated effects and disease-associated phosphorylation changes of nephrin in the animal injected with human anti-nephrin IgG. Finally, we precipitated IgG bound in rabbit glomeruli and applied IP followed by Western blotting for nephrin, revealing that bound antibodies were specific for nephrin (Figure 1I).

In summary, these experiments demonstrate that patient-derived IgG containing anti-nephrin autoantibodies induces the development of a podocytopathy with minimal changes upon transfer to another species with sufficient antigen crossreactivity. We acknowledge, as a limitation of our study, that, due to the generally low levels of anti-nephrin autoantibodies in affected patients, we were not able to purify the nephrin-specific IgG fraction for passive transfer. However, the specificity for nephrin of glomerular bound IgG in the anti-nephrin rabbit, in combination with the induction of nephrin phosphorylation in this rabbit, strongly suggests anti-nephrin autoantibodies as a causative factor in the development of minimal change disease. In conclusion, our discovery allows for a pathogenesis-based disease classification and strengthens the use of B cell–targeted therapies. It further provides a rationale to develop therapies targeting pathogenic autoantibodies and autoreactive B cells for patients with an anti-nephrin–associated podocytopathy (4).

## Supplementary Material

Supplemental data

Unedited blot and gel images

Supporting data values

## Figures and Tables

**Figure 1:**
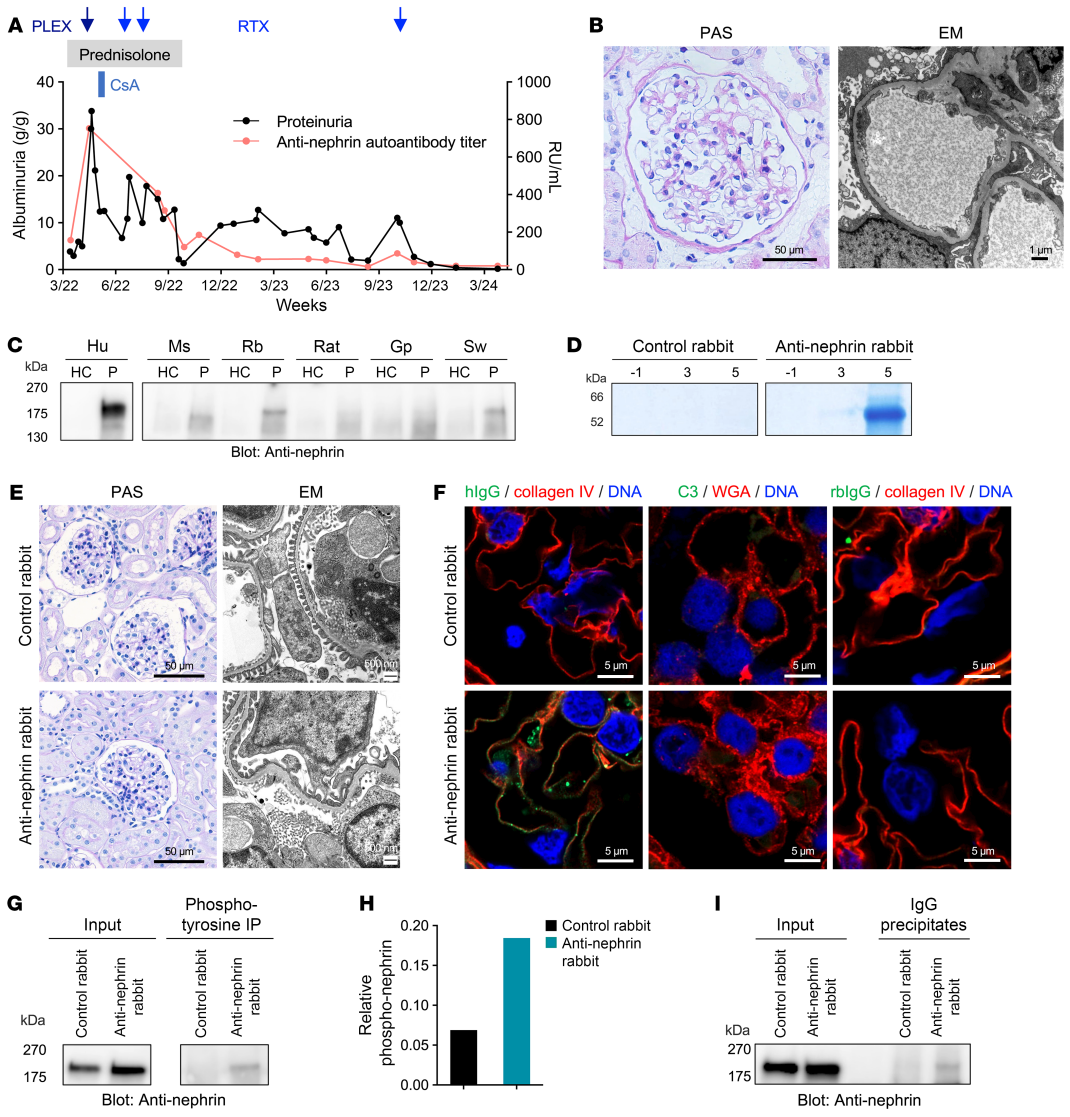
Anti-nephrin autoantibodies cause a podocytopathy with minimal change lesions. (**A**) Disease course of a patient with anti-nephrin–associated MCD. Albuminuria given as urinary albumin-to-creatinine ratio (g/g). PLEX, plasma exchange; CsA, cyclopsporine A; Rtx, rituximab. (**B**) Representative periodic acid-Schiff (PAS) staining (left) and electron microscopy (EM, right) of the patient. Scale bars: 50 μm (PAS) and 1 μm (EM). (**C**) Western blot of immunoprecipitates (human (hu), mouse (ms), rabbit (rb), rat, guinea pig (gp), and swine (sw) glomerular extracts with serum from the patient (P) or from a healthy control (HC) individual). (**D**) Coomassie-blue stainings of urine samples from rabbits before (day –1) and after (days 3 and 5) the transfer of human IgG containing anti-nephrin autoantibodies or control IgG. (**E**) PAS stainings (left) and EM images (right) of rabbits 5 days after IgG transfer. Scale bars: 50 μm (PAS) and 500 μm (EM). (**F**) Representative immunofluorescence stainings for human IgG (hIgG) in colocalization with collagen IV (left), complement C3 in colocalization with wheat germ agglutinin (WGA, middle), and rabbit IgG (rbIgG) in colocalization with collagen IV (right) of kidneys from rabbits 5 days after IgG transfer. Panels are enlargements of the boxed areas in Supplemental Figure 2C. Scale bars: 5 μm.(**G**) Western blot of rabbit glomerular extracts (left) and of immunoprecipitates (rabbit glomerular extracts and anti-phosphotyrosine antibody,right). (**H**) Relative signal intensity given as the ratio of tyrosine-phosphorylated nephrin and input nephrin signal in (**G**). (**I**) Western blot of rabbit glomerular extracts (left) and of rabbit nephrin precipitated by human IgG present in glomerular extracts (right).

## Version 1 01/16/2025

In-Press Preview

## Version 2 03/03/2025

Electronic publication

## References

[1] Hengel FE (2024). Autoantibodies targeting nephrin in podocytopathies. N Engl J Med.

[2] Watts AJB (2022). Discovery of autoantibodies targeting nephrin in minimal change disease supports a novel autoimmune etiology. J Am Soc Nephrol.

[3] Shirai Y (2024). A multi-institutional study found a possible role of anti-nephrin antibodies in post-transplant focal segmental glomerulosclerosis recurrence. Kidney Int.

[4] Seifert L (2024). An antigen-specific chimeric autoantibody receptor (CAAR) NK cell strategy for the elimination of anti-PLA2R1 and anti-THSD7A antibody-secreting cells. Kidney Int.

