# Supplementary material for: Passive transfer of patient-derived anti-nephrin autoantibodies causes a podocytopathy with minimal change lesions
Source: J Clin Invest. 2025 Jan 16;135(5):e186769. doi: 10.1172/JCI186769 (PMC11870725; doi:10.1172/JCI186769)
Supplement: Supplemental data [file jci-135-186769-s128.pdf]

## Supplementary material

### Table of contents

**Case report**
**Supplementary methods**
- Generation of recombinant human nephrin
- Quantitative IP/ELISA assay
- IgG purification
- Glomeruli isolation
- Glomerular extract preparation
- Immunoprecipitation
- IgG elution and immunoprecipitation from rabbit glomeruli
- Animal experiments
- Tissue stainings
- Sex as a biological variable
- Statistics
- Study approval
- Data availability
- Author contributions
- Acknowledgments

**Supplementary references**

**Supplementary figures**
- Supplementary figure S1
- Supplementary figure S2

## Case report

In April 2022, a 71-year-old female developed a rapid-onset nephrotic syndrome and was diagnosed with MCD by kidney biopsy (Figure 1A and 1B in the main part). The patient was initially treated with prednisolone 1 mg/kg body weight (Figure 1A in the main part, grey bar). In addition, she received supportive treatment with statins, diuretics, and anticoagulants. Further workup upon persistent massive nephrotic syndrome included positron emission tomography–computed tomography, which revealed a type A thymoma, followed by minimally invasive thymectomy in May 2022 and adjuvant radiotherapy. Severe albuminuria and hypoalbuminemia (<15 g/dl) persisted despite these measures, prompting us to start plasma exchange (PLEX, Figure 1A in the main part, dark blue arrow). The patient was additionally started on cyclosporine A (CsA, Figure 1A in the main part, blue bar) with no beneficial short-term effect and then switched to rituximab (RTX, Figure 1A, blue arrows), leading to complete peripheral blood B cell depletion. Three months after the first dose of RTX, albuminuria improved to the subnephrotic range in October 2022 (Figure 1A in the main part, black line). By that time, we had established anti-nephrin autoantibody measurements (as described previously (1) as well as described below) and found high autoantibody levels at the time of exacerbating nephrotic syndrome in May 2022 and low autoantibody levels at the time of partial remission (Figure 1A in the main part, red line). The improvement in albuminuria and anti-nephrin levels was closely followed by relapsing albuminuria two months after prednisolone tapering in December 2022. Albuminuria gradually decreased over the time course of 8 months to approximately 2 g/g albumin-to-creatinine ratio in August 2023. Another relapse occurred in October 2023 in parallel with the repopulation of B cells and an increase in anti-nephrin autoantibody levels, which was successfully treated with a single dose of RTX (Figure 1A, blue arrow).

## Supplementary methods

Generation of recombinant human nephrin

The ectodomain of human nephrin (NCBI reference sequence: NM_004646.4, AA A25-G1037) in the eucaryotic expression vector pXLG was generously provided by Matthias Wilmanns (EMBL, Hamburg). The protein carried a C-terminal Twin-Strep-tag and an 8x polyhistidine-tag for downstream purification and analysis, which was done as described in detail before (see supplementary information) (1). Protein quality and sample purity was validated by Western blot and/or Coomassie staining. The protein concentration was determined using a spectrophotometer (Biozym Scientific).

Quantitative anti-nephrin IP/ELISA assay

Anti-nephrin autoantibodies were quantified by a two-step procedure of immunoprecipitation followed by quantification of immunoprecipitated recombinant nephrin as described earlier (1). 60 µl patient or control serum (human or rabbit) was mixed with 150 ng of recombinant human nephrin in radioimmunoprecipitation assay buffer (RIPA buffer, 150 mM NaCl, 1% NP40, 0.5 % deoxycholate, 0.1% sodium dodecyl sulfate, 50 mM Tris pH 7.4), incubated over night at constant rotation and exposed to MabCaptureC™ High Capacity Protein A Resin (Thermo Fisher Scientific) for 2 hours. The resin was washed using Pierce™ Spin Columns (Thermo Fisher Scientific) twice in RIPA buffer followed by 4 washes in phosphate buffered saline (PBS) with 0.2% Tween 20 (PBS-T) and once in PBS. Protein elution was achieved by acidification using IgG elution buffer (Thermo Fisher Scientific) at pH 2.8 and neutralization with 1M Tris pH 9. Subsequently, immunoprecipitated recombinant Twin-Strep-tagged nephrin was quantified using a Streptactin-coated microplate (IBA Lifesciences GmbH) incubated with the eluate for 2 hours at 20 °C, washed four times with TBS-T (Sigma-Aldrich) and incubated with anti-nephrin antibody (polyclonal sheep anti-nephrin antibody, R&D Systems; AF4269, diluted 1:1000 in post coat buffer with 0.05% Tween 20) overnight on a rocking platform at 4°C. Wells were washed four times and incubated with 100 µl of HRP-conjugated donkey anti-sheep IgG (1:10,000, Jackson ImmunoResearch, AB_234071)for one hour at 20 °C. Wells were washed again before application of TMB ELISA peroxidase substrate solution (Avia Systems Biology) for 5 min at 20 °C, followed by acidification using 100 µl of 1 mol/L $H_3PO_4$-solution to stop the substrate reaction. The absorbance at 450 nm was determined using an ELISA reader (EL808, Bio-Tek instruments). Quantification was done in duplicates and relative units (RU/ml) were determined in regard to a standard curve of serial dilutions of recombinant human nephrin.

IgG purification

NAB protein G columns (Thermo Fisher Scientific) were equilibrated with PBS and patient plasmapheresis sample or control serum was applied. Columns were washed with PBS twice, followed by antibody elution by acidification using IgG elution buffer (Thermo Fisher Scientific) at pH 2.8 and neutralization with 1M Tris pH 9. Eluates were concentrated (vivaspin 20 50K, Sartorius) and buffer exchanged to PBS (PD-10 desalting columns with Sephadex G-25 resin, cytiva). Sample quality and

purity was controlled by Western blot/Coomassie staining and antibody concentration was determined using a spectrophotometer (Biozym Scientific). C

Glomeruli isolation

Glomeruli isolation was performed on ice. Mouse glomeruli isolation was performed as described before (1). Mouse kidneys were perfused with tosyl-activated dynabeads M-450 (Invitrogen) in Hanks' Balanced Salt Solution (HBSS) with calcium and magnesium (Thermo Fisher Scientific), minced using a scalpel, homogenized with a compact tissue homogenizer (Minilys, Bertin Technologies), repetitively strained using tissue dissociation sieves (300 µm, 100 µm pore size) and washed with cold HBSS. After centrifugation at 500 g for 5 min at 4°C the pellet was resuspended in cold HBSS. Glomeruli were separated using a magnetic particle concentrator (Invitrogen) and repetitively washed with HBSS supplemented with 0.5 % BSA (Carl Roth). Glomeruli were counted and pelleted by centrifugation at 4,000 g for 5 min at 4 °C. Supernatant was discarded and the glomerular cell pellets were snap-frozen and stored at -80 °C until further use. For rat, rabbit, guinea pig, swine and human glomeruli isolation, kidneys were perfused with PBS (not possible for human kidney samples), fibrous tissue removed, and kidney cortex minced with a scalpel. Minced kidney tissue was sieved through pre-wetted tissue dissociation sieves (150 µm and 106 µm for rat and rabbit kidney, 150 µm and 90 µm for guinea pig kidney, 300 µm and 150 µm for pig kidney, 150 µm for human kidney), and extensively washed with PBS enriched with 0.05% human or bovine albumin (CSL Behring or Carl Roth, respectively). The resulting flow-through was washed again with albumin-enriched PBS and cleaned from small tissue components using a 53 or 90 µm cell strainer. Glomeruli were rinsed, resuspended, counted and pelleted at 1200 g for 15 min at 4 °C. Supernatant was discarded and the glomerular cell pellets were snap-frozen and stored at -80 °C until further use.

Glomerular extract preparation

Glomerular extracts were prepared by lysis of isolated mouse, rabbit, rat, guinea pig, swine, or human glomeruli in tissue lysis buffer (20 mM Tris/Cl pH 7,4, 150 mM NaCl, 2 mM $CaCl_2$, 1% Triton X100, 1% inhibitor cocktail plus X100 (Carl Roth) or 1% Halt™ Protease und Phosphatase Inhibitor Cocktail and EDTA (Thermo Fisher Scientific)). Lysates were sonicated for 20 seconds at 30% power for 4 times (Sonopuls™ HD 2070 homogenisator (Bandelin Electronic)), left on ice for 1 h, and centrifuged at

20,000 x g for 10 min at 4 °C. The lysate supernatant was used immediately or stored at -20 °C until further use.

Immunoprecipitation

Glomerular lysates of different species or recombinant human nephrin were used for immunoprecipitation with 25 µl patient/control serum or purified IgG in tissue lysis buffer. After overnight incubation at constant rotation at 4 °C, MabCaptureC™ High Capacity Protein A Resin (Thermo Fisher Scientific) was added and incubated for 2 hours at constant rotation at 4 °C. The resin was washed twice in lysis buffer, three times in phosphate buffered saline (PBS) with 0.2% Tween 20 (PBS-T) and once in PBS. Protein elution was achieved under denaturing conditions using 2.5x Laemmli buffer (300 mM Tris-HCl, pH 6.8, 50% glycerol, 10% SDS, 1% bromophenol blue, 250 mM DDT) and incubation at 95 °C for 10 min. Samples were electrophoresed in gradient gels (4-15% TGX, BioRad) by SDS-PAGE and transferred to methanol-soaked PVDF membranes (EMD Millipore) under semi-dry conditions. Membranes were blocked with 5% dry milk in Tris-buffered saline with 0.05% Tween (TBS-T) at 20 °C for 3 hours and incubated with primary antibody (polyclonal sheep anti-nephrin antibody, R&D Systems; AF4269) diluted 1:400 in Superblock blocking buffer (Thermo Fisher Scientific) at 4 °C overnight. After washing three times with TBS-T, membranes were incubated with HRP-conjugated donkey anti-sheep IgG (Jackson ImmunoResearch, AB_234071) in 5% dry milk TBS-T (1:10,000) for 1 hour at 20 °C and washed 3 times again. Finally, membranes were incubated with chemiluminescent substrate (SuperSignal West Pico or Femto; Thermo Fisher Scientific) and imaged by incremental luminescence detection with Amersham Imager 600. Immunoprecipitation of phosphorylated tyrosine residues and subsequent detection of precipitated nephrin was performed as described before (1).

IgG elution and immunoprecipitation from rabbit glomeruli

Glomeruli of the anti-nephrin and control rabbit were isolated as described above. Next, glomeruli were lysed in tissue lysis buffer (20 mM Tris/Cl pH 7,4, 150 mM NaCl, 2 mM $CaCl_2$, 1% Triton X100, or 1% Halt™ Protease und Phosphatase Inhibitor Cocktail and EDTA (Thermo Fisher Scientific), sonicated, left on ice and centrifuged as described above. The lysate supernatant included glomerular proteins as well as human IgG previously bound to rabbit glomeruli and was subsequently incubated overnight at constant rotation at 4 °C to allow for (re-)formation antibody-antigen complexes potentially separated

during glomeruli lysis. Next, MabCaptureC™ High Capacity Protein A Resin (Thermo Fisher Scientific) was added and incubated for 2 hours at constant rotation at 4 °C. The resin was washed twice in lysis buffer, three times in phosphate buffered saline (PBS) with 0.2% Tween 20 (PBS-T) and once in PBS. Protein elution was achieved under denaturing conditions using 2.5x Laemmli buffer (300 mM Tris-HCl, pH 6.8, 50% glycerol, 10% SDS, 1% bromophenol blue, 250 mM DDT) and incubation at 95 °C for 10 min. Samples were electrophoresed and immunoblotted as described above.

Animal experiments

Female New Zealand White rabbits were purchased from Envigo Charles River Laboratories and housed in groups. Rabbits had free access to food and standard animal chow. Animals of the same age were randomly assigned to immunization. A total of 480 mg of purified patient IgG containing anti-nephrin IgG or control IgG was transferred to the animals intravenously, subdivided into three injections (day 0, day 1, day 3). Metabolic cages were used for urine collection and weight was monitored daily. Plasma samples were taken at baseline and on the day of sacrifice. Animals were sacrificed after an observation period of five days after immunization for blood and organ collection. Animals were monitored for clinical signs of (immune-mediated) immediate reaction towards the infused IgG and complement consumption was measured by Western blotting of rabbit sera and detection with anti-C3 (Genetex, GTX72994, 1:1000), anti-C5 (Complement Tech, A220, 1:2000) and HRP-coupled anti-goat-IgG (1:10,000, Jackson ImmunoResearch). Urinary protein was measured using Coomassie-blue stainings under non-reducing conditions and normalized to urinary creatinine determined by Jaffe. Urine amount was normalized to 7 µg of creatinine with the exception of the day 5 urine from the anti-nephrin IgG-injected rabbit, which was normalized to 1 µg of creatinine to account for the high level of proteinuria and avoid protein overload on the gel.

Tissue stainings

Periodic acid Schiff (PAS) staining, immunofluorescence staining and electron microscopic analysis was performed as described before (1). For Immunofluorescence staining, the following antibodies were used: wheat germ agglutinin (WGA)-rhodamin (1:400; Vector-Laboratories), C3 (FITC goat anti-C3, 1:50; Cappel 55500), Collagen IV (goat anti-Collagen IV, 1:400, Southern Biotech, 1340-01). The following fluorochrome-conjugated secondary antibodies were used (all 1:200, Jackson

ImmunoResearch Laboratories): anti-goat IgG AF488 (705-545-147), anti-goat IgG Cy3 (705-165-147), anti-rabbit IgG AF488 (711-545-152), anti-rabbit IgG Cy3 (711-165-152), anti-human IgG Cy2 (709-225-149).

Sex as a biological variable

Our study involved human material from a female patient and female rabbits; this choice was based on availability and not on expected sex-dimorphic effects. Sex was not considered as a biological variable, and we expect the findings to be relevant for all sexes.

Statistics

No statistical testing was applied in this proof-of-concept study.

Study approval

The study was approved by the local ethics committee of the Chamber of Physicians in Hamburg and conducted in accordance with the ethical principles stated by the Declaration of Helsinki. The patient gave written informed consent. Animal experiments were performed according to national and institutional animal care and ethical guidelines and were approved by the Veterinarian Agency of Hamburg and the local animal care committee (registration number N074-2023).

Data availability

Data of the findings of this study are included in the main article and supplementary material (see Supporting Sata Values file). Personally identifiable patient values are not given due to ethical reasons. Further information is available from the corresponding authors upon request.

Author contributions

NMT and TBH initiated and jointly supervised the study. FH and NMT designed experiments. FH, SD and OK performed experiments and generated data. FH, SD, OK, NMT, and TBH analyzed data. JE and TZ supported acquisition and interpretation of data. FH and NMT wrote the first draft of the manuscript. All authors vouch for the data and the analyses. All authors decided to publish the paper.

There were no agreements concerning confidentiality of the data between the authors and any institution.

Acknowledgements

We thank Sigrid Harendza and Maida Mahmud for clinical expertise and sample acquisition and Marie-Luise Schröder, Jutta Dammann, and Sarah Hewald for extraordinary support and animal care. We thank Friedrich Koch-Nolte for support with protein production in HEK293-6E cells.

This work was supported by the Deutsche Forschungsgemeinschaft as part of the collaborative research center 1192 (to FEH, TBH and NMT) and as part of the Emmy Noether Research Group (TO1013 to NMT), by the BMBF (STOP-FSGS- 01GM2202A to TBH), by the Else-Kröner Fresenius Foundation (Else Kröner-Clinician-Scientist-Program iPRIME to FEH), by the Else Kröner-Fresenius-Stiftung and the Eva Luise und Horst Köhler Stiftung (Clinician-Scientist-Program RECORD to FEH), and by the European Research Council (CureFSGS to TBH and AUTO-TARGET to NMT).

## Supplementary references

1. Hengel FE, et al. Autoantibodies Targeting Nephrin in Podocytopathies. *New England Journal of Medicine.* 2024;391(5):422-33.

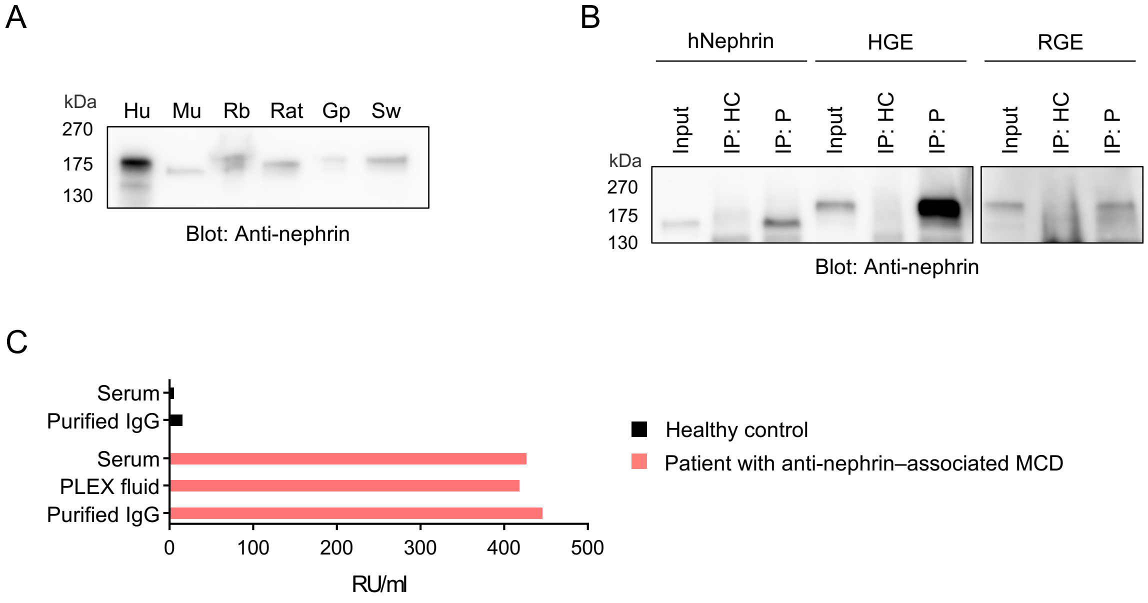


**Supplementary Figure 1. Reactivity of human autoantibodies with nephrin.** (**A**) Western blot of human (hu), murine (mu), rabbit (rb), rat, guinea pig (gp), and swine (sw) glomerular extracts with a commercial anti-nephrin antibody. These glomerular extracts were used for the immunprecipitation experiments with patient serum that are shown in Figure 1C. (**B**) Western blot of immunoprecipitates (purified IgG from the serum of a healthy control (HC) individual or plasma exchange (PLEX) fluid from the patient (P) with anti-nephrin–associated MCD and recombinant human nephrin (hNephrin), human glomerular extract (HGE) or rabbit glomerular extract (RGE) detected with a nephrin-specific antibody. The IgG purified from the P, but not the HC, reacted with hNephrin as well as nephrin present in HGE and RGE. (**C**) Anti-nephrin antibody titers measured by immunoprecipitation followed by ELISA using serum and purified IgG from a HC as well as serum, PLEX fluid, and IgG purified from the PLEX fluid from the P with anti-nephrin–associated MCD.

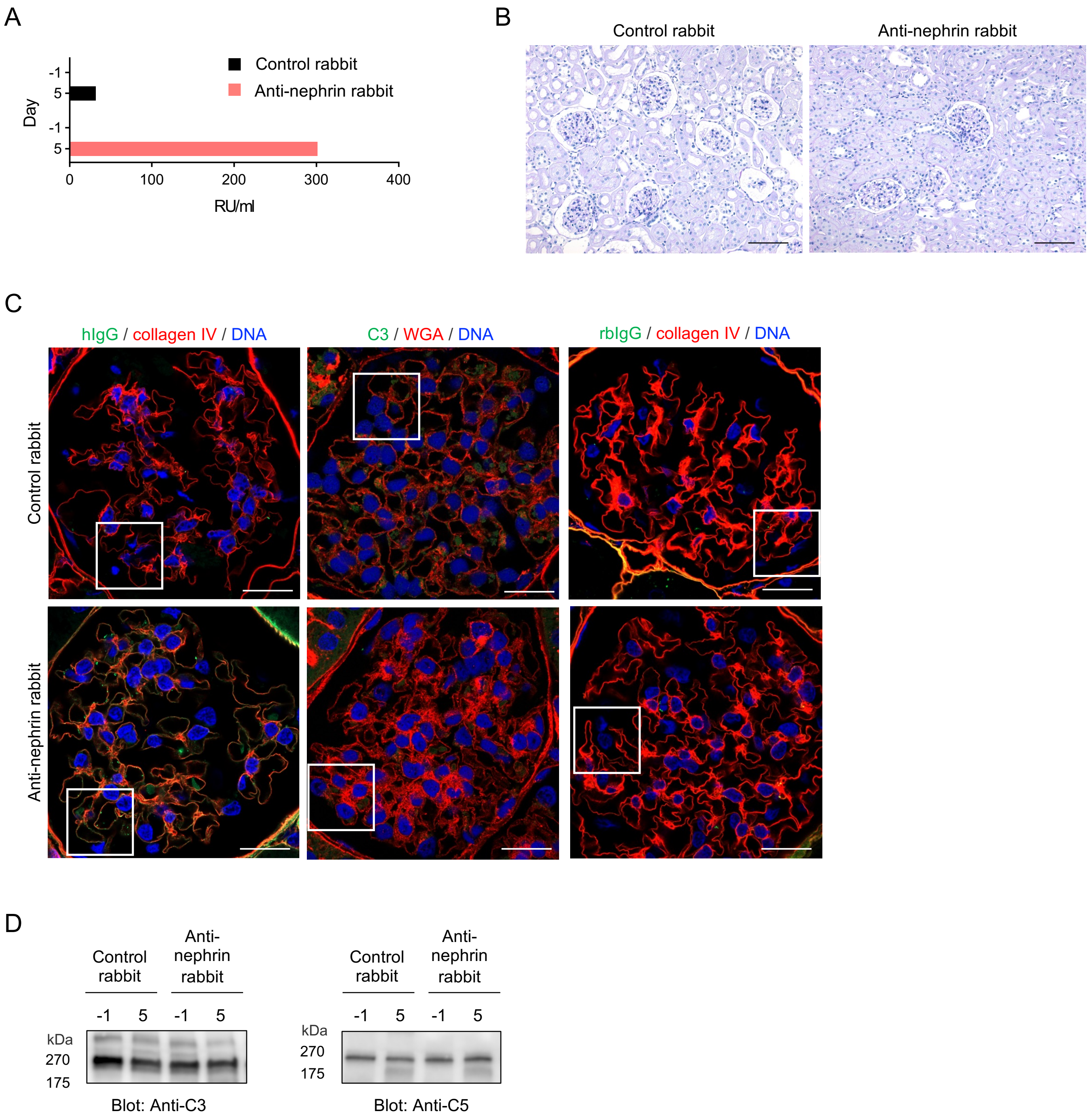


**Supplementary Figure 2. Passive transfer of human IgG into rabbits.** (**A**) Anti-nephrin antibody titers measured by immunoprecipitation followed by ELISA in rabbits before (day -1) and after (day 5) the transfer of human IgG containing anti-nephrin autoantibodies (red) and control (black). (**B**) Periodic acid Schiff (PAS) stainings of rabbit kidneys 5 days after passive IgG transfer. Animals did not show morphological alterations such as hypercellularity, thickening of the glomerular basement membrane or glomerual sclerosis. Scale bars denote 100 µm. Images show the same specimen as in Fig. 1E at different magnification. (**C**) Representative immunofluorescence stainings for human IgG (hIgG) in co-localization with collagen IV (left), complement C3 in co-localization with wheat germ agglutinin (WGA, middle), and rabbit IgG (rbIgG) in co-localization with collagen IV (right) of kidneys from rabbits 5 days after passive IgG transfer. Scale bars denote 20 µm. (**D**) Western blots of rabbit sera before (day -1) and after (day 5) passive IgG transfer with C5- (left) and C3-specific (right) antibodies. Results do not show evidence for complement consumption upon passive transfer of purified human IgG.
