# Supplementary material for: Passive transfer of patient-derived anti-nephrin autoantibodies causes a podocytopathy with minimal change lesions
Source: J Clin Invest. 2025 Jan 16;135(5):e186769. doi: 10.1172/JCI186769 (PMC11870725; doi:10.1172/JCI186769)

Full unedited blots for Figure 1C:
Anti-Nephrin Autoantibody Reactivity of Patient IgG

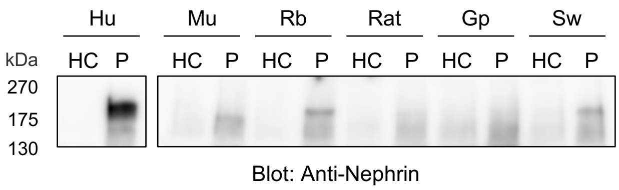

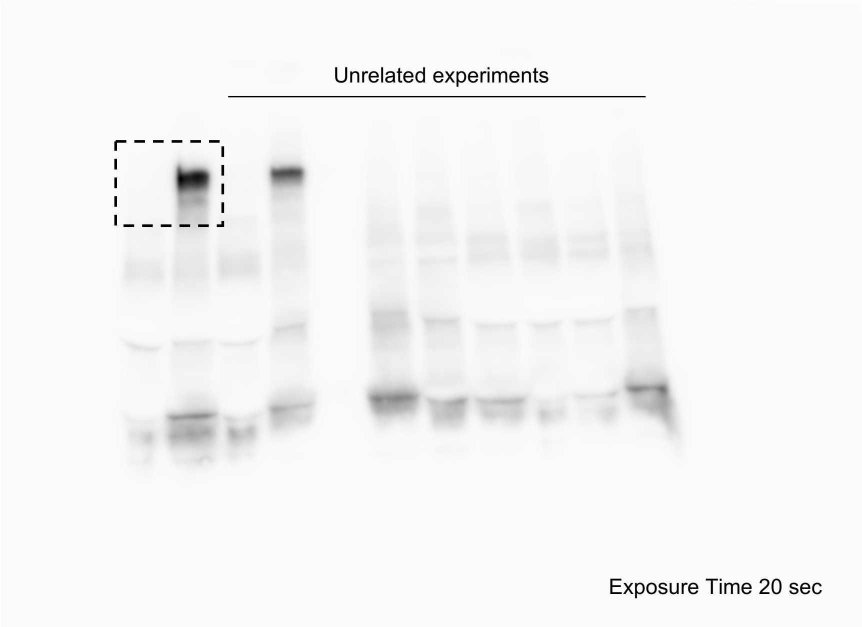

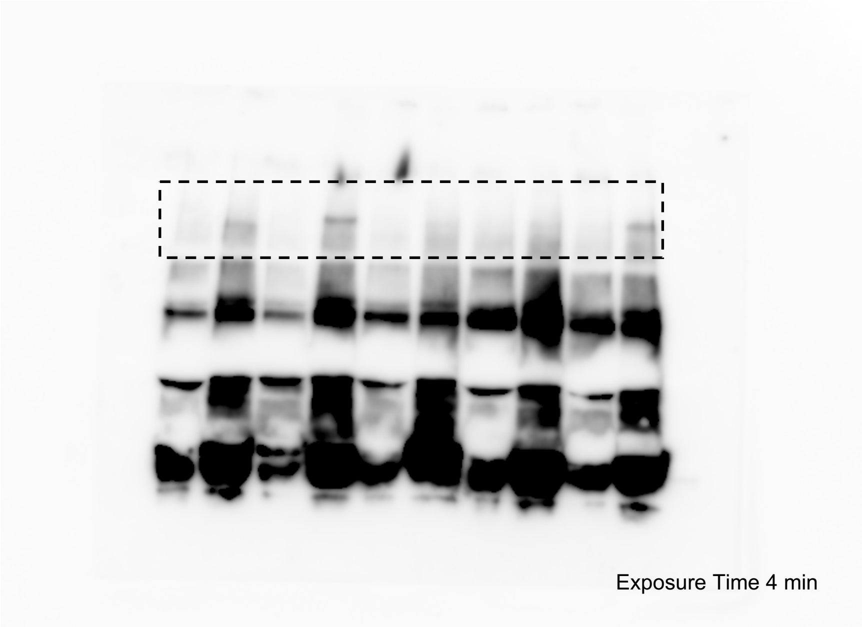

Full unedited gel for Figure 1D:
Proteinuria in Rabbits IgG Transfer

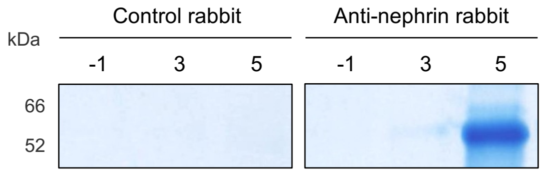

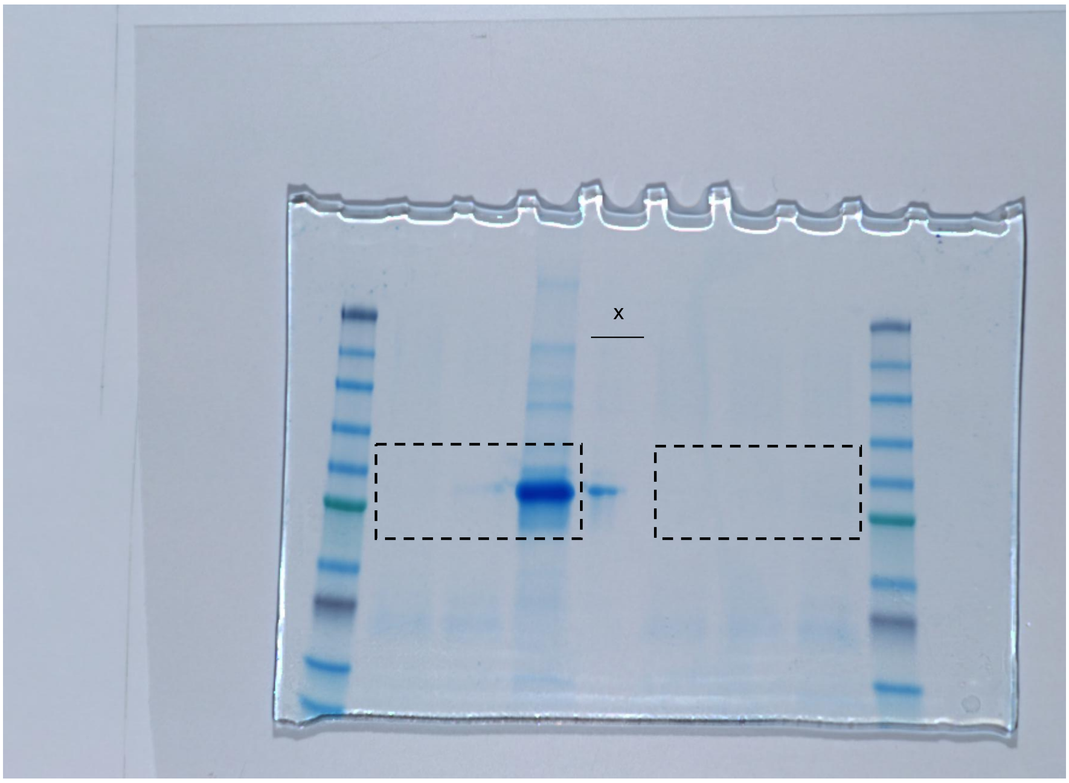

Full unedited blot for Figure 1G:
Quantification of nephrin phosphorylation upon passive transfer of purified IgG

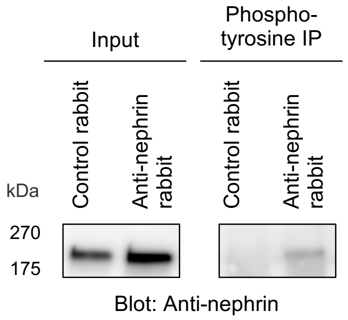

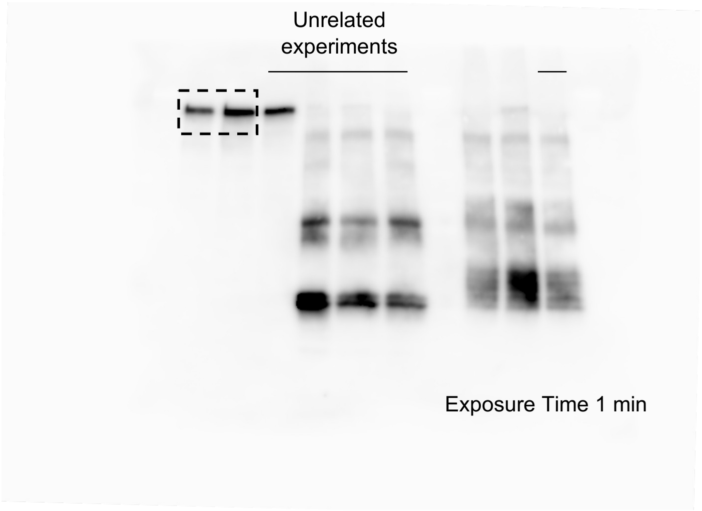

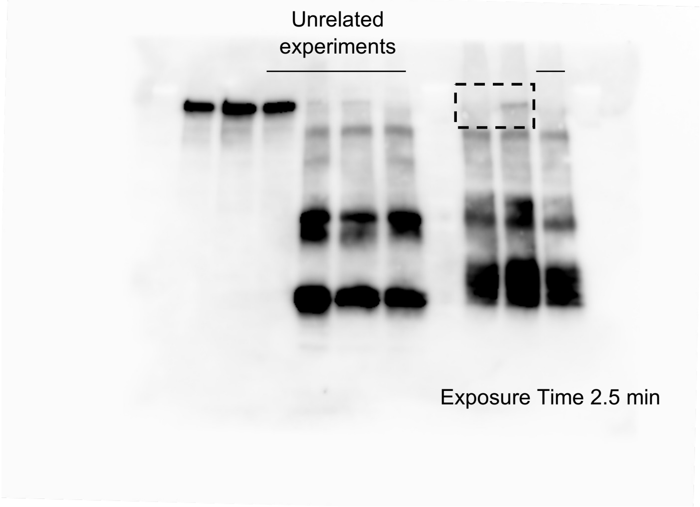

Full unedited blot for Figure 1I:
Elution of intraglomerularily bound human IgG and subsequent Immunoprecipitation of rabbit nephrin

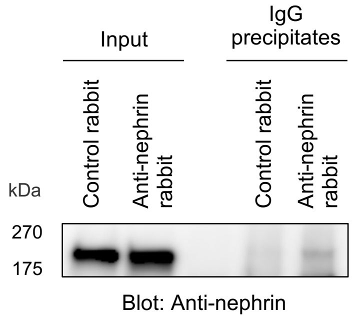

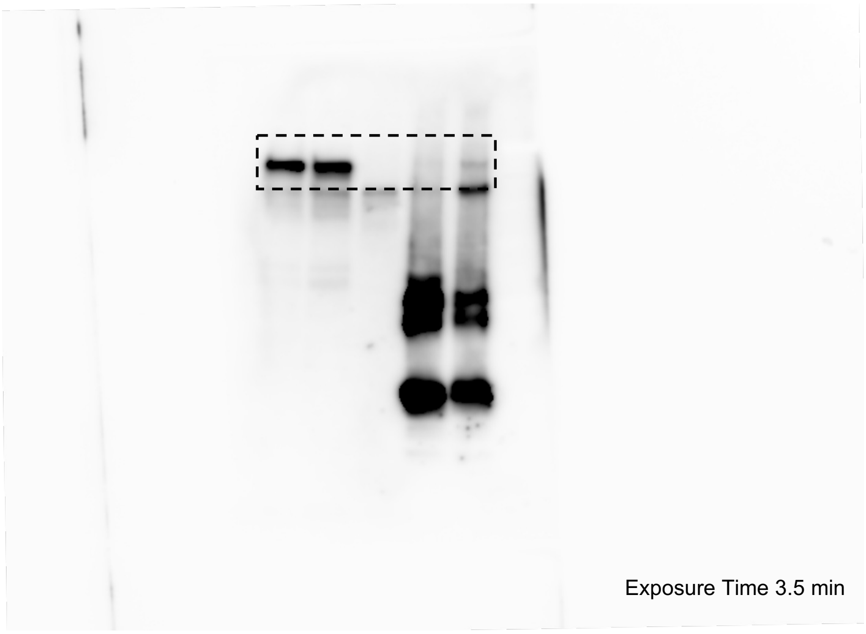

Full unedited blot for Supplementary Figure 1A:
Glomerular Extracts Used for Immunoprecipitation

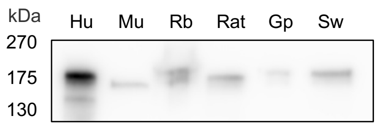

Blot: Anti-Nephrin

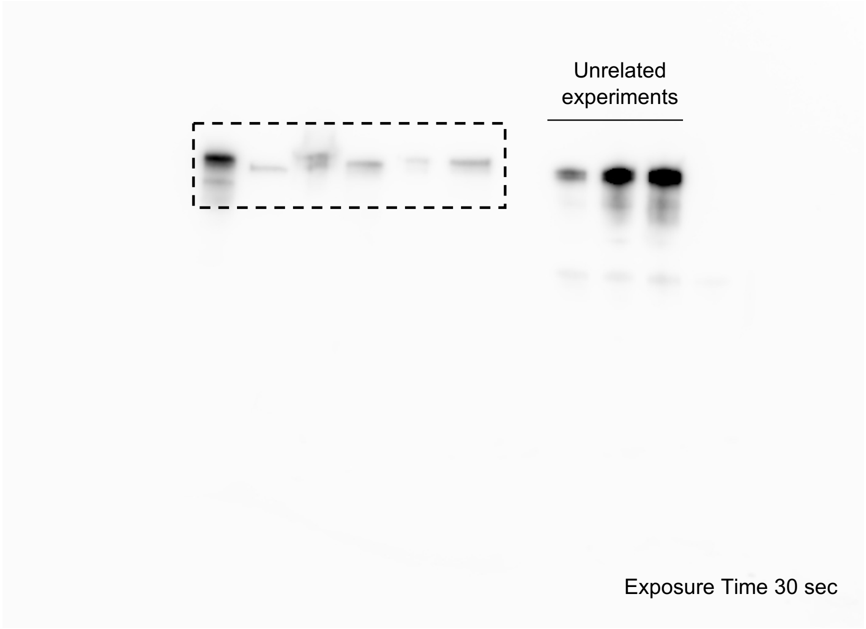

Full unedited blot for Supplementary Figure 1B: Immunoprecipitated recombinant human nephrin and nephrin from human glomerular extract (HGE) and rabbit glomerular extract (RbGE) using purified IgG

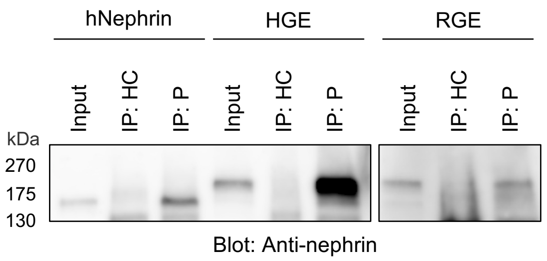

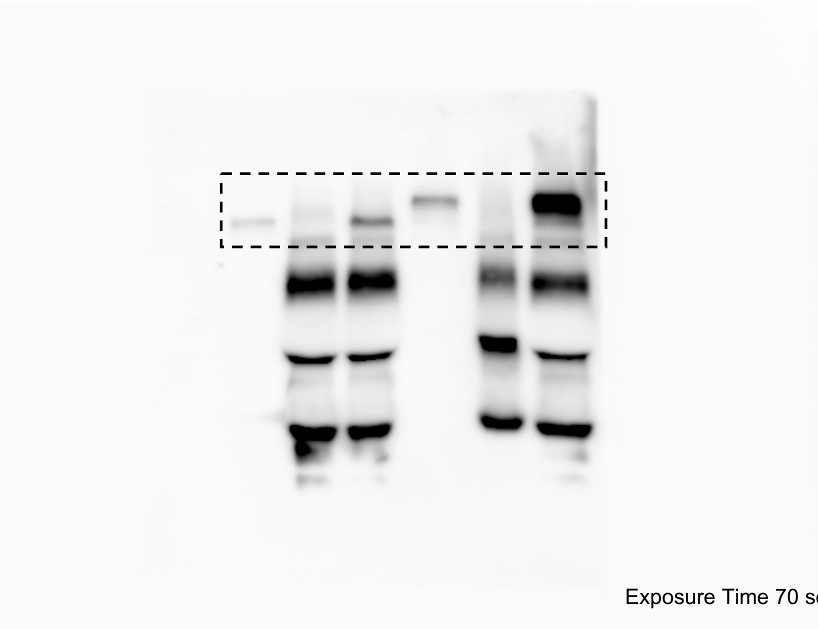

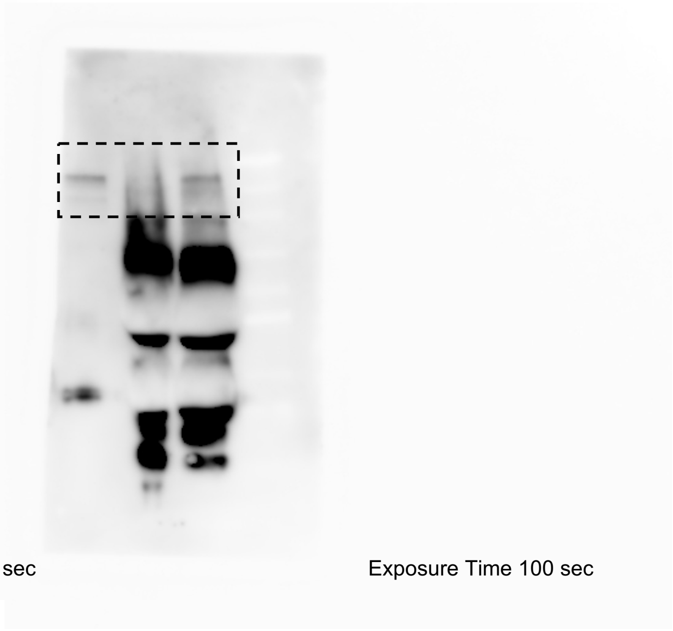

Full unedited blot for Supplementary Figure 2D:
Rabbit serum complement C5 and C3

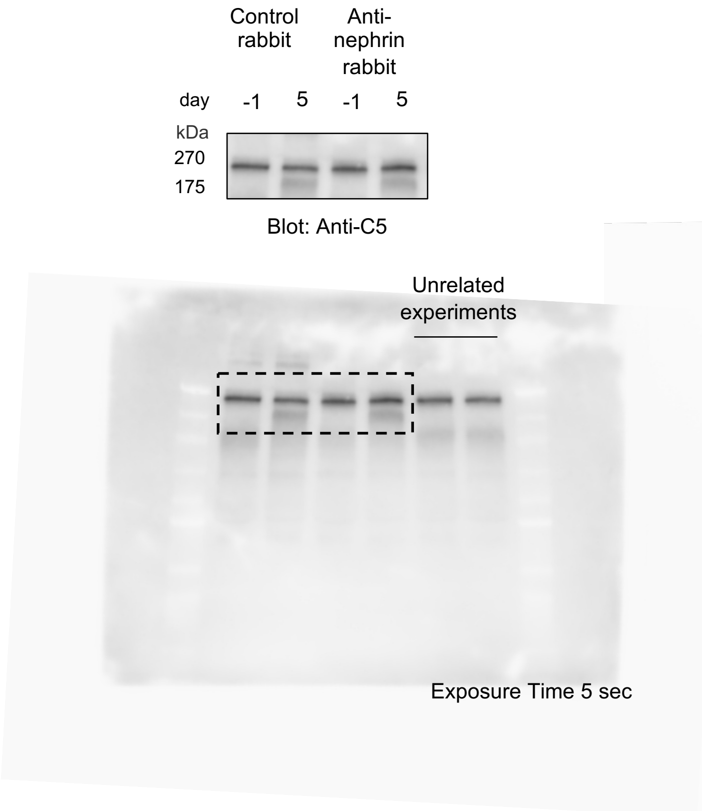

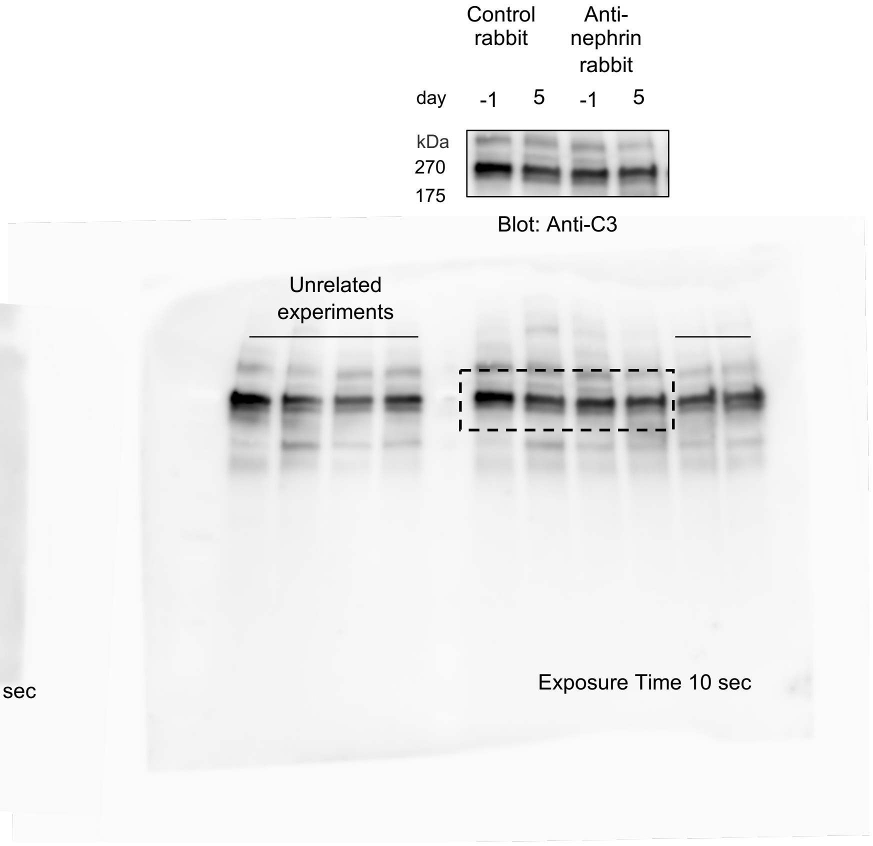

Supplement: Unedited blot and gel images [file jci-135-186769-s129.pdf]
